# Measurement of small droplet aerosol concentrations in public spaces using handheld particle counters

**DOI:** 10.1063/5.0035701

**Published:** 2020-12-01

**Authors:** G. Aernout Somsen, Cees J. M. van Rijn, Stefan Kooij, Reinout A. Bem, Daniel Bonn

**Affiliations:** 1 Cardiology Centers of the Netherlands, Amsterdam, The Netherlands; 2 Van der Waals-Zeeman Institute, Institute of Physics, University of Amsterdam, Amsterdam, The Netherlands; 3 Department of Pediatric Intensive Care, Emma Children’s Hospital, Amsterdam University Medical Centers, Location AMC, Amsterdam, The Netherlands

## Abstract

We measure aerosol persistence to assess the risk of transmission of severe acute respiratory syndrome coronavirus 2 (SARS-CoV-2) in public spaces. Direct measurement of aerosol concentrations, however, has proven to be technically difficult; we propose the use of handheld particle counters as a novel and easily applicable method to measure aerosol concentrations. This allows us to perform measurements in typical public spaces, each differing in volume, the number of people, and the ventilation rate. These data are used to estimate the relation between the aerosol persistence time and the risk of infection with SARS-CoV-2.

## INTRODUCTION

The World Health Organization, in its recent scientific brief,^1^ has highlighted the possible role of aerosols in the transmission of severe acute respiratory syndrome coronavirus 2 (SARS-CoV-2)^1–4^ and stated that “much more research is needed given the possible implications of such route of transmission.”^1^ This is particularly relevant for public spaces where the risk of aerosol transmission of SARS-CoV-2 is highest. Direct measurement of aerosol concentrations, however, has proven to be technically difficult,^5–7^ hampering such research. We validate the use of handheld particle counters as a novel and easily applicable method to measure aerosol concentrations. Particle counting has been used before (see, e.g., Ref. 8), but the method has not been validated; the main challenges are to distinguish aerosols from background dust and the fact that due to external conditions such as temperature and relative humidity, the persistence and dispersion of both small and large droplets may evolve over time.^9–13^ To demonstrate the usefulness of our novel method, we perform measurements in typical public spaces that can play a role in aerosol transmission of SARS-CoV-2, each differing in volume, the number of people, and the ventilation rate. These data are used to estimate the relation between the aerosol persistence time and the risk of infection with SARS-CoV-2.

## METHODS

Aerosol concentration is often measured using the laser sheet diffraction technique, in which the number of pixels that light up is a measure for the number and volume of the droplets.^4,5^ However, this technique can only be operated by highly specialized personnel and, because of laser safety issues, only in laboratory settings. Using this technique as the standard, we validate a novel method using a handheld particle counter (Fluke 985, Fluke B.V. Europe, Eindhoven, The Netherlands), which is frequently used for air quality assessment and overcomes most of the above-mentioned drawbacks of the laser sheet diffraction technique. The specifications of the Fluke device are six size channels of 0.3 *µ*m, 0.5 *µ*m, 1.0 *µ*m, 2.0 *µ*m, 5.0 *µ*m, and 10.0 *µ*m. Air is pumped into the device at a flow rate of 2.83 l/min and flows through the detection region where a 90 mW laser beam of 775 nm–795 nm wavelength illuminates the dust or aerosol particles, and the scattered and diffracted light from these is detected with a counting efficiency of 50% for the 0.3 *µ*m channel and 100% for particles in all the other channels. The accuracy and reproducibility of these measurements are both 1%. The Fluke instrument is the one we discuss here, but similar results were obtained with other particle counters, notably Lighthouse and Trotec instruments. As a reference, we used a SprayScan® (Spraying Systems, Glendale Heights, IL, USA) laser sheet to track the aerosols by filming the laser light scattering of the aerosol droplets directly using a CCD camera and image analysis software. For each measurement, the aerosol concentration is determined by correcting for the background (measured for ∼8 min), consisting of dust particles.

## VALIDATION

**FIG. 1.**
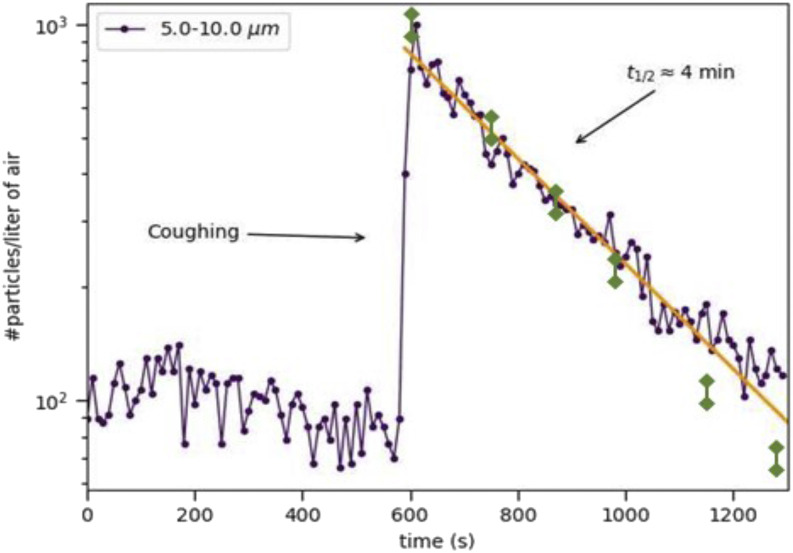
Concentration of airborne particles of diameters 5.0 *µ*m–10.0 *µ*m as a function of time as measured using a handheld particle counter (purple symbols) and the laser sheet diffraction technique^4^ in a poorly ventilated space of volume 8 m^3^. The other channels of the particle counter yield comparable results. The arrow indicates the moment of coughing, coinciding with a sharp increase followed by an exponential decay, with a half-life of roughly 4 min. The yellow line is a guide to the eye. The green data points are the reference data from the laser sheet technique published previously in Ref. 4.

First, we validate the particle counting method by comparing the results with those obtained previously using the laser sheet scattering and laser diffraction techniques.^4^ The typical validation scenario is that of a single person coughing once inside a poorly ventilated restroom of volume 8 m^3^. The results for both techniques in this specific example are shown in Fig. 1. We find that coughing leads to the generation of an amount of aerosol particles an order of magnitude above the background level of the particle counter; both techniques next reveal that the number of aerosols per liter of air decreases exponentially in time, with a time constant of ∼4 min (Fig. 1). This was done in this and three other rooms, and the correlation coefficient between the results of the two techniques was always better than 0.97.

**FIG. 2.**
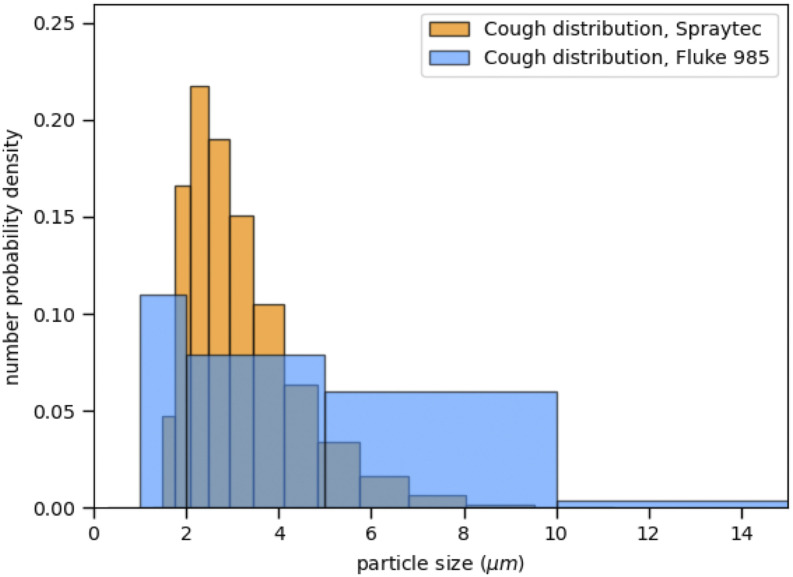
Number particle size distributions measured by laser diffraction (yellow bars) compared with the distribution inferred from the particle counter (blue bars). The limited number of channels of the particle counter makes for a crude distribution, but the two are compatible.

Figure 2 shows the size distribution of the particles as obtained by the two techniques. The comparison is more difficult here, as the handheld particle counter has less channels to separate ranges of particle sizes as the laser diffraction technique used in Ref. 5 (Malvern Spraytech®). The resulting drop size distribution is a compound Gamma distribution, as explained in Refs. 5 and 14. The distribution obtained with the Fluke instrument is much coarser due to the smaller number of channels. However, the results are compatible with comparable mean values of 4.9 ± 1.7 and 3.6 ± 0.4, respectively, for the Fluke and the Spraytech results; importantly, it also shows that the particle counter covers the whole range of aerosol sizes produced by coughing. We also find that the results obtained using the handheld particle counter were reproducible to within 10%. We conclude that the particle counter technique is, indeed, a reliable method to determine aerosol concentrations as a function of time and as well to give a rough indication of the size distribution of droplets.

## APPLICATION TO PUBLIC SPACES

We now use the technique to characterize a wide range of real-world public spaces, each selected as a typical example of a certain type of public space (Table I). In these public spaces, we measure all aerosols resulting from breathing, speaking, coughing, and sneezing by people present (1–25 at the time of the measurements). Background measurements indicate that the dust generated by the people moving around is mostly (>98%) contained in the first two channels of the particle counter (diameters between 0.3 *μ*m and 0.5 *μ*m), out of the range of the characteristic diameter of aerosols produced by breathing, speaking, coughing, and sneezing.^4^ They are, therefore, not considered when evaluating the aerosol density. Separate from the measurements involving multiple people producing aerosols, we also investigate the decrease in aerosol concentration over time by generating a known quantity of artificial aerosols using a specially designed spray nozzle as used in Ref. 4, which is known to produce aerosols of the same size distribution as respiratory droplets resulting from coughing (i.e., between 1 *μ*m and 10 *μ*m with a maximum at 4 *μ*m). The risk assessment was done and explained in detail in Ref. 5 and is based on the persistence time of the aerosols: the analysis is done for the same particle size distribution with different persistence times. In Table I, we summarize the results, indicating the number of people involved, the air change rate per hour (ACH) of the ventilation system used, and the measured droplet concentration half-times. All measurements were done at least in five different locations at the measurement site so that differences in the airflow and proximity of walls were averaged out. The results for a given site were found to be identical to within 5%. In the public spaces investigated by us, aerosol concentrations are ∼20 to more than 100 times lower in all ventilated public spaces compared to the poorly ventilated restroom used for calibration measurements. In addition, in a public elevator and in a poorly ventilated living room, aerosol concentrations are high. The characteristic times for a 50% decrease in aerosol concentration are on the order of 1 min in well-ventilated spaces, compared to 4 min–5 min in the poorly ventilated restroom, elevator, and living room. This is due to both the air renewal (given by the ACH) and further dilution by dispersion throughout the space and therefore depends on both the ACH and the absolute size of the given space (Table I). All the ACH values given in Table I were from the installation companies.

**TABLE I.** Aerosol concentrations and persistence times in different public spaces characterized by the number of people present, volume, and rate of ventilation. In all but the club scenario where aerosols were artificially generated, the aerosol origin is from speaking, coughing, and sneezing of the people. Spaces were sampled, which gave us permission to do so—these were mainly well-ventilated spaces in modern buildings. Each space was typically sampled in five different places. Estimated viral load based on calculations.

		Air change per		50% decrease			Covid-19 infection
Space	Size (m^3^)	hour (h^−1^)	Aerosol origin	(min)	Aerosol part/l	RNA copies/l	risk
Gym	2 000	5–15	25 visitors	1	<10	<1	Low
Train	150	0–5	20 visitors	2	210	<21	Low
Meeting room	30	10	4 visitors	1	45	<5	Low
Night club	2 000	5–15	Artificial	1	<10	<1	Low
Car	3	5–20	2 visitors	0.5	20	<2	Low
Airport	12 000	5–15	∼100 visitors	1	<10	<1	Low
Restaurant	120	8	25 visitors	1	248	<25	Low
Restroom	8	∼1	1 visitor	4	7716	<772	Intermediate
Office space	50	10	5 visitors	1	35	<4	Low
Unventilated living room	80	∼1	4 visitors	5	5214	<520	Intermediate
Elevator	8	∼1 to 4	2 visitors	5	4350	<435	Intermediate

## DISCUSSION

This study shows that the particle counter technique is a reliable method to investigate aerosol concentrations and their evolution in time. Using this easily applicable method, aerosol concentrations can be measured in any public space, which is important to determine the risk of aerosol transmission of SARS-CoV-2 and to evaluate the impact of risk reducing measures (i.e., improving ventilation).

Aerosol persistence times in the tested spaces are relatively short due to adequate space ventilation. Current standards for the air change rate by mechanical room ventilation depend on the occupation of the room(s) but roughly vary between 2 air changes/h and 15 air changes/h. An air change rate of 10 times/h means that for every 6 min, the given space has received fresh air of a volume similar to that of the space. Our measurements of aerosol persistence suggest that a half-life of the aerosol concentration of 1 min or 2 min minimizes the aerosol concentration and is achieved for air change rates in excess of 10 air changes/h. Using the half-times as measured by us, we calculate that the decrease in the number of aerosol particles after these 6 min will vary between 50% and 100%, depending on the ventilation method and the size of the public space. We also note that since the Fluke instrument has its own pump, the results do not notably depend on the air flow in a room since the flow through the instrument is dominated by its pump.

When translating our findings to practical risk assessments in the context of SARS-CoV-2 transmission by aerosols, we conclude that the risk of transmission via airborne aerosols is low in all but the restroom, elevator, and unventilated living room scenarios. The reason for this is twofold: First, good ventilation significantly decreases the density of aerosols in a short time (Table I). Second, the number of viral particles in very small aerosol drops is low. Sputum droplets from COVID-19 patients carry typically between 10^4^ and 10^9^ RNA copies/ml.^15^ This implies between 0.001 and 100 RNA copies per thousand aerosol drops.^15,16^ The minimum infectious dose for SARS-CoV-2 has not been reported; the severity of COVID-19 is, however, believed to be proportional to the dose of the initial inoculum, implying that transmission by aerosols may lead to relatively milder symptoms.^5,16^ In our risk analysis, we assume that exposure to less than 10^3^ microdroplets (corresponding to less than 100 RNA copies) imposes a low risk, between 10^3^ and 10^5^ microdroplets (corresponding to 100–10 000 RNA copies) imposes an intermediate risk, and more than 10^5^ microdroplets (corresponding to more than 10 000 RNA copies) imposes a high risk of transmission.^5,15^ Further research on the transmissibility of the virus is needed to validate this assumption and possibly correct these risk values. Our measurements together with the risk analysis of Refs. 5 and 15, however, underline the importance of good ventilation and suggest that health authorities can advise a minimal ventilation rate to minimize the probability of SARS-CoV-2 transmission in public spaces.

## CONCLUSION

This study demonstrates a novel aerosol measurement method that is easily implemented in different environments. With reasonable assumptions on the viral load and infectivity, this allows for a rough estimate of the probability of aerosol transmission of SARS-CoV-2 following Refs. 5 and 16. To reduce the spread of such infections, healthcare authorities should consider this method to evaluate the ventilation of public spaces, especially in spaces, such as hospital and dentistry settings, where aerosolization is common.

## DATA AVAILABILITY

The data that support the findings of this study are available from the corresponding author upon reasonable request.
